# An occupational pipe leak poisoning

**DOI:** 10.3389/fphar.2025.1514565

**Published:** 2025-07-29

**Authors:** Xiangxing Zhang, Tianzi Jian, Mengdi Shi, Chen Wang, Xiangdong Jian, Qilu Li

**Affiliations:** ^1^ Department of Poisoning and Occupational Diseases, Emergency Medicine, Qilu Hospital, Cheeloo College of Medicine, Shandong University, Jinan, Shandong, China; ^2^ Department of Occupational and Environmental Health, School of Public Health, Cheeloo College of Medicine, Shandong University, Jinan, Shandong, China; ^3^ Department of Hematology, Qilu Hospital, Cheeloo College of Medicine, Shandong University, Jinan, Shandong, China; ^4^ The Hospital of Shandong University, Cheeloo College of Medicine, Shandong University, Jinan, Shandong, China

**Keywords:** dimethylacetamide, 4,4′-diaminodiphenyl ether, peripheral neuropathy, toxic encephalopathy, methemoglobinemia, toxic liver damage

## Abstract

**Introduction:**

Dimethylacetamide (DMAC) is used as a solvent in the dye, leather, and acrylic manufacturing industries. However, it is associated with significant risks of liver and nerve damage. 4,4’-Diaminodiphenyl ether (4,4’-ODA) is a crucial precursor for synthesizing curing agents and dyes but can disrupt the methemoglobin reduction system, resulting in methemoglobinemia.

**Methods:**

This paper reports an incident involving a plant pipeline leak on May 10, 2024, which primarily contained a DMAC solution and trace amounts of 4,4’-ODA. The patient’s occupational history, onset time of clinical symptoms, physical examination results, laboratory data, and treatment course were analyzed, and follow-up evaluation was performed to monitor the patient’s long-term health status.

**Results:**

Two patients exhibited clinical symptoms, including respiratory failure, methemoglobinemia, hemolytic anemia, and acute liver injury due to dermal and respiratory exposure. They were successfully treated and discharged. Notably, Patient 1 was diagnosed with toxic encephalopathy 16 days post-exposure, while Patient 2 developed peripheral neuropathy 58 days post-exposure.

**Discussion:**

This study aimed to elucidate the clinical characteristics associated with this type of poisoning. Moreover, the incident calls for enhanced safety measures in industries using DMAC and 4,4’-ODA to prevent similar poisoning events.

## 1 Introduction

Dimethylacetamide (DMAC) (chemical formula, C_4_H_9_NO) is a colorless and transparent liquid that is soluble in both water and organic solvents such as benzene, alcohol, and ether. It is used as a solvent in various processes, including the production of synthetic resins and fibers (Nomiya et al., 2025). However, it can be absorbed through the skin and has significant irritative effects on the eyes, skin, and mucousmembranes. It may exert detrimental effects on both the nervous system and hepatic function (Antoniou et al., 2021). Notably, no cases of toxic encephalopathy or peripheral neuropathy have been reported.

4,4′-Diaminodiphenyl ether (4,4′-ODA) (chemical formula, C_12_H_12_N_2_O) is a white or light-yellow crystalline substance that exhibits slight solubility in water but dissolves readily in ethanol. It has an LD_50_ value of 725 mg/kg for rats and is an aromatic amine compound (Singh et al., 2014). It is used as a crucial raw material for the synthesis of hardening agents and dyes. Exposure to 4,4′-ODA via inhalation or dermal absorption may lead to poisoning.

This study details an incident involving a pipeline leak at a factory on 10 May 2024, primarily containing DMAC along with potential residual amounts of 4,4′-ODA. Two patients were exposed through cutaneous contact and respiratory routes, resulting in methemoglobinemia accompanied by hemolytic anemia, acute liver injury, peripheral neuropathy, and toxic encephalopathy.

## 2 Clinical data

### 2.1 General patient information and factory production processes

Patient 1 was a 39-year-old man with 10 years of service. He had a history of good health prior to presentation. Patient 2 was a 64-year-old man with 16 years of service. He had a history of hypertension. They were an electric welder at the same leather factory. Both individuals were employed as electric welders at the same leather manufacturing facility, where they participated in pipe repair and equipment installation tasks. Their work environment typically involved outdoor settings without the use of protective gear such as hard hats, goggles, gloves, or respirators. They maintained an average work schedule of 8 hours per day for 28 days each month.

The factory produced 4,4′-ODA, which is used in the production of plastic films. The production process involved included the following: DMAC was added to the solution kettle to facilitate the reduction reaction of dinitrodiphenyl ether with hydrogen. Dinitrodiphenyl ether and activated carbon were introduced into the manhole, where the mixture was heated with steam and stirred. Once the dinitrodiphenyl ether was completely dissolved, the material was filtered into the reduction kettle using nitrogen pressure. After replacing the nitrogen, the reduction reaction was initiated by introducing hydrogen while agitating the mixture. After the reaction, the material was pressed through a filter using nitrogen. The filtrate was then transferred to the distillation kettle, where DMAC and water were distilled and condensed into a storage tank. The distillation residue was then pushed into the sublimation kettle with nitrogen, where the heating gas was condensed and crystallized in the crystallization chamber to yield crude 4,4′-ODA. Additionally, the event pipeline stored the DMAC solution for recycling after the reaction, which may also contain residue from 4,4′-ODA. The pipeline of this equipment had been used for 6 years without repair. The waste liquid had also been stored in the pipeline.

### 2.2 Poisoning incidents and treatment

At 8 a.m. on 10 May 2024, the two patients were conducting pipeline maintenance in the old factory area. Upon opening the blue sheet of the pipeline, a brown-red liquid containing rust sprayed out, accompanied by a pungent odor. The liquid sprayed onto the patients’ bodies, affecting their hair, face, eyes, and other areas. Both patients reported a burning sensation and observed blackened skin where the liquid had contacted them. They were washed after 20 min of exposure.

#### 2.2.1 Patient 1

On the way to the hospital (approximately 40 min after the incident), Patient 1 experienced dizziness, confusion, shallow breathing, limb twitches, and dyspnea, with symptoms gradually worsening. Upon admission, he was delirious and comatose. His pupils were large and round, measuring approximately 3 mm in diameter, with a slow light reflex. Cyanosis was observed in the skin of his face, lips, extremities, and nail beds. His heart rate was 100 beats/min and regular, with no pathological murmurs detected in any valve area. His blood pressure was 88/48 mmHg, and his respiratory sounds were thick in both lungs, with no dry rales present. The arterial blood gas analysis revealed the following: pH, 7.382 (7.350–7.450); lactic acid, 2.96 mmol/L (0.50–1.70); pCO_2_, 37.7 mmHg (35.0–45.0); pO_2_, 61 mmHg (80–105); and methemoglobin, 43.6%. Immediate treatment included tracheal intubation and ventilator-assisted ventilation. Three hours later, a repeat arterial blood gas analysis revealed the following: pH, 7.294 (7.350–7.450); and lactic acid, 10.48 mmol/L (0.50–1.70). The patient received intravenous injections of methylene blue (20 mg, twice a day) and dexamethasone (10 mg), along with liver protection and blood purification treatments. The laboratory test results for Patient 1 are presented in Table 1. On May 11, the toxicology test indicated a DMAC concentration of 0.530 μg/mL in the blood (detection was performed using liquid chromatography-tandem mass spectrometry (LC-MS/MS). The patient developed hemolytic anemia, with significantly increased white blood cell count, liver function markers, and creatine kinase levels, all of which progressively worsened. The patient was transferred to our hospital on the sixth day after the poisoning for further diagnosis and treatment.

**TABLE 1:** Laboratory test results of the patient 1 at different time-points following hospital admission.

Biochemical blood indicators	Local hospital							Our department						
	Normal values	Day1	Day2	Day3	Day4	Day5	Day6	Normal values	Day6	Day8	Day12	Day19	Day28	Day60
WBC(×10^9^/L)	**3.5–9.5**	23.85	27.07	25.98	28.12	24.30	33.55	**3.5–9.5**	29.23	31.33	27.27	6.83	5.14	7.73
NEU(%)	**40–75**	99.8	90.9	87.8	86.4	—	83.2	**40–75**	86.10	86.30	85.40	71.50	56.60	59.70
ALT (IU/L)	**0–41**	53	50	35	27	—	37	**0–35**	43	37	76	147	54	38
AST (IU/L)	**0–37**	29	52	40	26	—	23	**14–36**	68	31	28	36	21	21
TBIL (μmol/L)	**5.1–19**	32.59	30.18	43.87	57.19	58.42	84.85	**3–22**	89	47.5	22.3	5.9	7.0	7.0
DBIL (μmol/L)	**1.7–6.8**	15.08	13.72	17.65	20.86	23.28	27.84	**0–5**	1	17.8	9.3	3.3	3.1	2.4
IBIL (μmol/L)	**2–22**	17.51	16.46	26.22	36.33	35.14	57.01	**0–19**	71	29.7	13.0	2.6	3.9	4.7
CK(U/L)	**24–190**	257.6	1,260	884.6	476.4	—	195.6	**30–135**	144	113	56	27	33	47
CK-MB(ng/mL)	**0–24**	20	41	28	16	—	9	**0.3–4**	1.20	0.5	1.8	1.9	0.9	0.8
BUN(mmol/L)	**1.7–8.3**	4.4	4.6	4.9	6.6	7.0	8.2	**2.5–6.1**	9.8	11.32	8.56	2.83	3.23	3.10
Cr (μmol/L)	**70–115**	88	76	76	63	54	62	**46–106**	73	82	61	65	76	87
IL-6 (pg/mL)	—	—	—	—	—	—	—	**0–7**	—	24.60	6.09	—	<2.00	—
RBC(×10^12^/L)	**4.3–5.8**	5.35	5.47	4.32	—	2.98	2.01	**4.3–5.8**	1.17	1.72	3.13	3.06	4.11	5.29
HGB (g/L)	**130–175**	158	156	122	—	82	75	**130–175**	62.0	59.0	103.0	99.0	129.0	154.0
DD-i (μ g/mL)	—	—	—	—		—	—	**<0.50**	2.99	5.56	5.16	1.00	0.72	0.36

WBC, white blood cells; NEU, neutrophils; ALT, alanine transaminase; AST, aspartate aminotransferase; TBIL, total bilirubin; DBIL; direct bilirubin; IBIL; indirect bilirubin; CK, creatine kinase; CK-MB, creatine kinase-MB; BUN, blood urea nitrogen; Cr, creatinine; IL-6, interleukin 6; RBC, red blood cells; HGB, hemoglobin; DD-i, D-dimer.

Values in bold represent the normal values of indicators for different hospitals.

**FIGURE 1:**
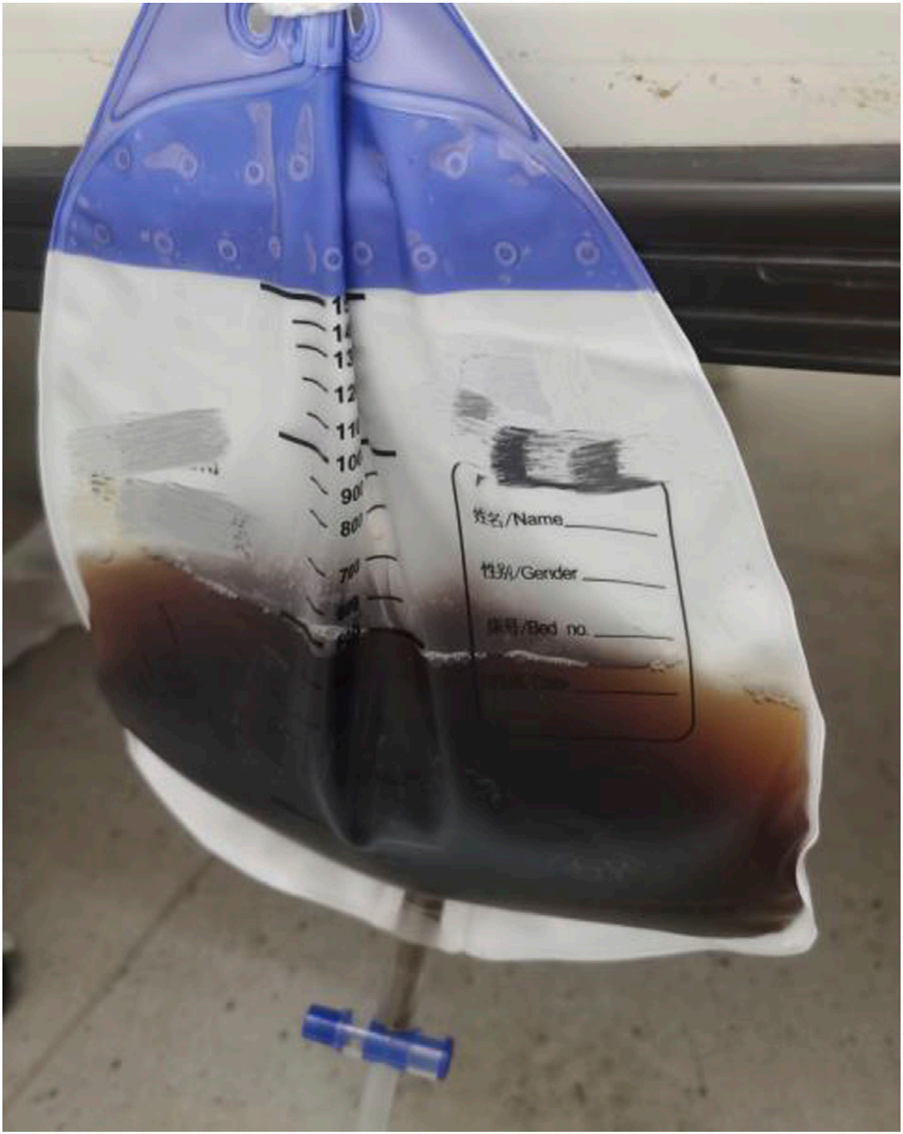
On the sixth day post-exposure to the toxins, the urine of Patient 1 exhibited a coloration similar to that of soy sauce.

**FIGURE 2:**
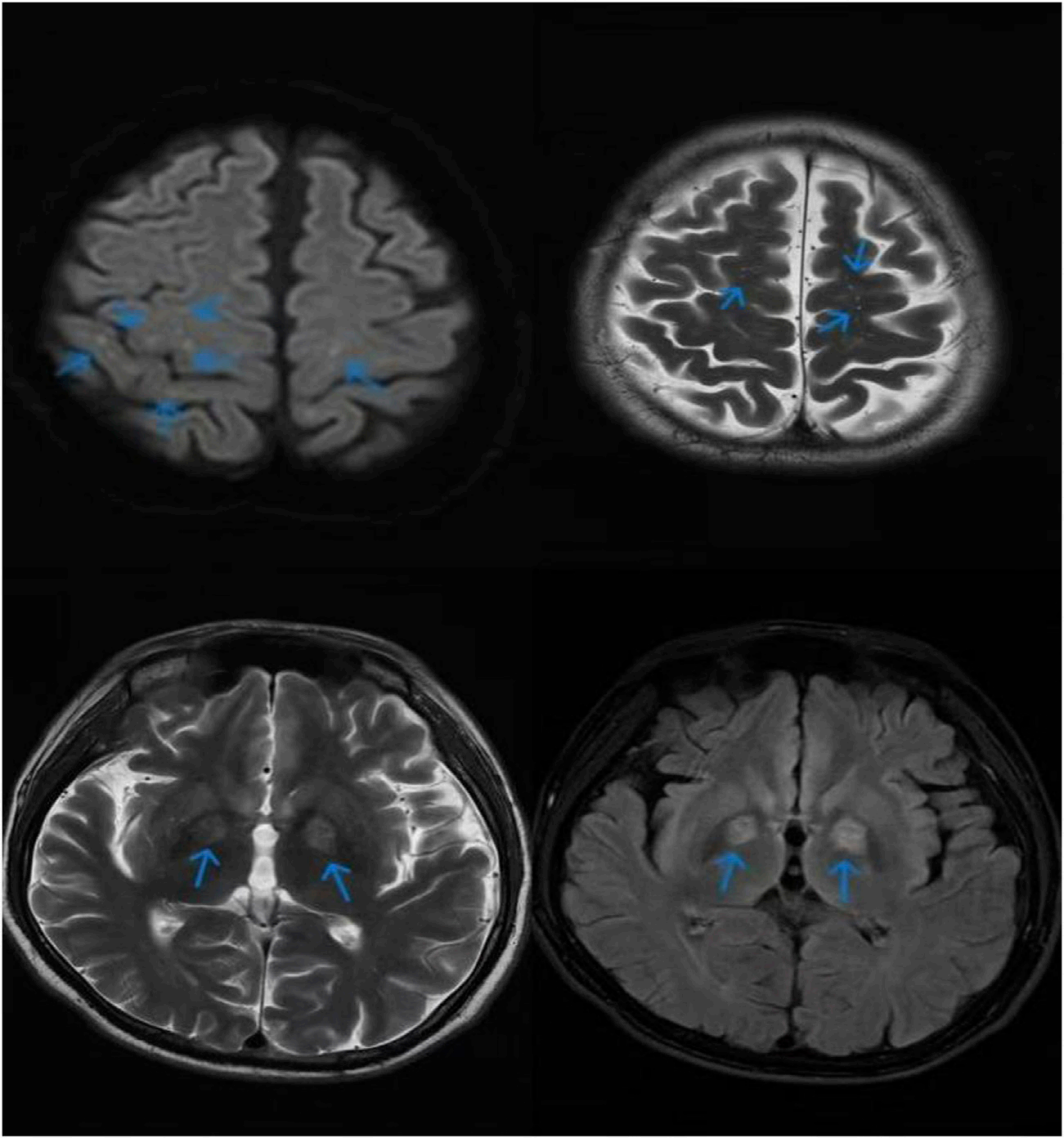
The cranial magnetic resonance imaging (MRI) result of Patient 1 revealed bilateral cerebral hemispheres beneath the cortex, with the internal capsule exhibiting a symmetrical distribution of spots and dots characterized by prolonged T1 and T2 signals. The T2 FLAIR sequence demonstrated hyperintensity, while diffusion-weighted imaging (DWI) indicated high signal intensity alongside multiple abnormal signals observed within the brain.

**FIGURE 3:**
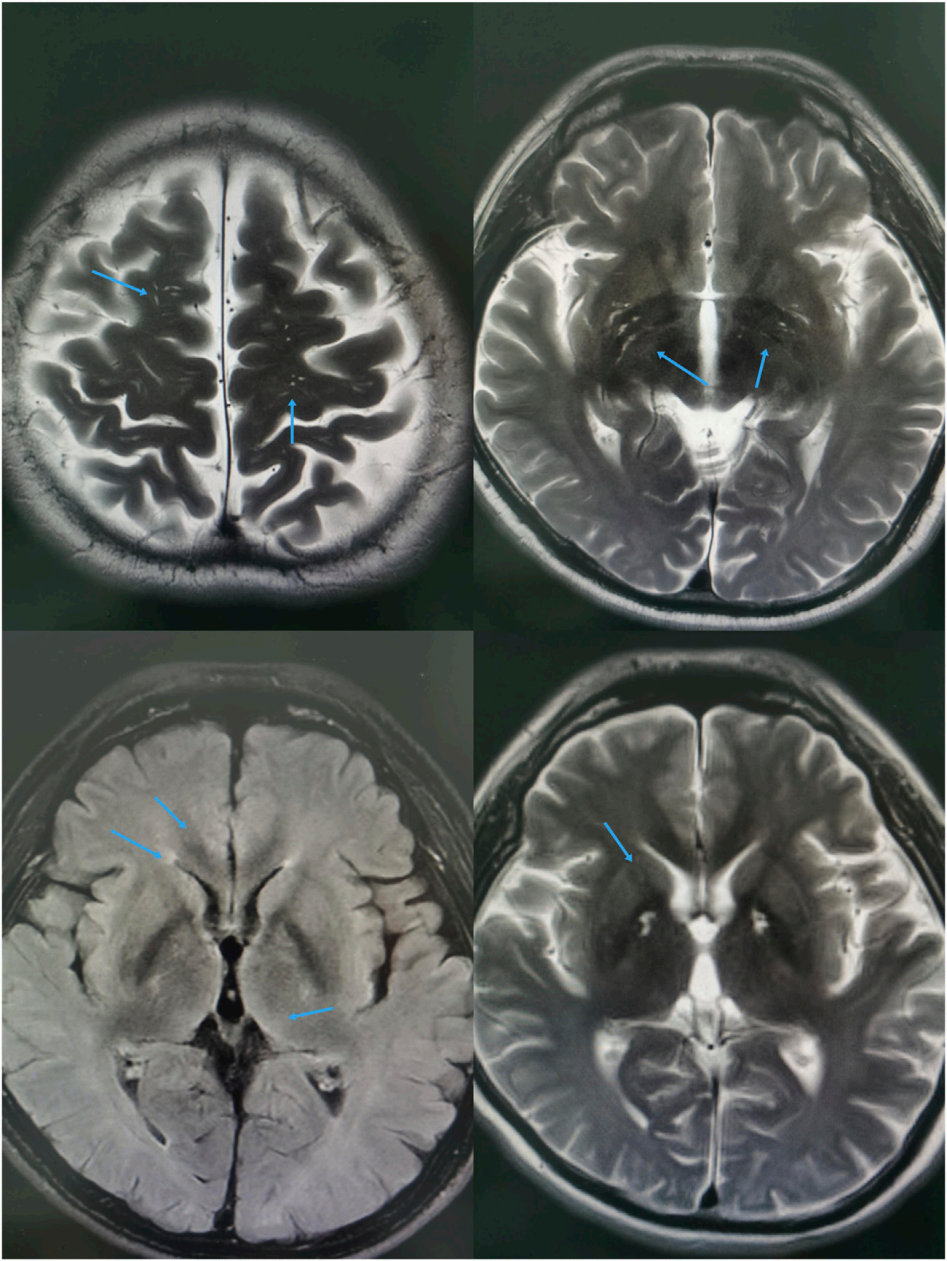
The MRI revealed white matter abnormalities in both cerebral hemispheres, with several long T_1_ and T_2_ signal lesions exhibiting symmetrical distribution within the basal ganglia. The T_2_ FLAIR sequence demonstrated hyperintensities, while diffusion-weighted imaging (DWI) indicated mildly elevated signals, alongside multiple abnormal findings throughout the brain.

Upon admission, he was sedated and on ventilator-assisted ventilation, displaying uncooperative behavior during the physical examination. He had yellowing of the skin and sclera, and his pupils were large and round, measuring about 3 mm in diameter with a dull light reflex. His heart rate was 84 beats/min, consistent and rhythmic, and his body temperature was 38.7°C. His urine was soy sauce-colored (Figure 1), with a volume of 1,200 mL. The arterial blood gas analysis revealed the following: pH, 7.527 (7.350–7.450); lactic acid, 2.1 mmol/L (1.0–1.8); and methemoglobin, 1.0% (0–3). His hemoglobin level was 62.0 g/L (130–175). The patient received two units of red blood cells, along with dexamethasone (10 mg), furosemide (20 mg), vitamin B_1_ (100 mg), as well as liver protective agents (polyene phosphatidylcholine), nerve nutrition, and blood purification treatments. On the seventh day after poisoning, the patient experienced sporadic limb convulsions, with the urine volume increasing to 1900 mL and its color intensity decreasing. Valproate was administered to control the convulsions, and two units of red blood cells were transfused alongside continued blood purification. On the ninth day, the patient’s consciousness improved, and the urine color returned to normal. By the 11th day, the patient’s condition had improved significantly. The tracheal tube was removed, and the yellowing of the sclera and skin was alleviated. On the 13th day, a chest computed tomography (CT) revealed a small amount of inflammatory changes in both lungs, fibroproliferative foci, and reduced density in the heart cavity, indicating anemia. No significant abnormalities were observed in the head and abdominal CT scans. However, the liver function markers remained elevated, prompting continued liver preservation therapy. On the 16th day, the patient reported numbness and weakness in his fingertips. Cerebral magnetic resonance imaging (MRI) revealed bilateral cerebral hemispheres under the cortex, and the internal sac showed symmetrical distribution of long T_1_ and T_2_ signals. The T_2_ FLAIR sequence demonstrated hyperintensity, while diffusion-weighted imaging (DWI) indicated high signal intensity alongside multiple abnormal signals observed within the brain, which was consistent with the manifestations of toxic encephalopathy (Figure 2). The patient received vitamin B_1_, mecobalamine, and other nerve nutrition treatments. By the 21st day, the patient’s condition had improved, and he was discharged from the hospital. On the 28th day, a follow-up chest CT showed improvement in the symptoms, and the range of abnormal cranial magnetic signals had reduced. However, on the 60th day of re-examination, the family reported decreased speech, and he reported persistent numbness and weakness in the left thumb. The cranial MRI revealed multiple abnormal signals in the brain (Figure 3). A few symmetrically distributed long T_1_ and T_2_ signals were observed in the white matter and basal ganglia areas of the bilateral cerebral hemispheres. The electromyography and electroencephalogram results showed no obvious abnormalities, and we continued to provide nutritional nerve therapy and other symptomatic treatments. Regular reviews indicated no obvious discomfort.

#### 2.2.2 Patient 2

On the way to the hospital, Patient 2 developed chest tightness, dyspnea, cyanosis of the mouth, and a pale face, with the symptoms progressively worsening. Upon admission, the patient exhibited a dull response, shallow breathing, and vague consciousness. His pupils were large and round, measuring approximately 3 mm in diameter, with a dull light reflex. His heart rate was 60 beats/min, and no pathological murmurs were detected in any valve area. His blood pressure was 120/70 mmHg. Moreover, the arterial blood gas analysis revealed the following: pH, 7.334 (7.350–7.450); lactic acid, 1.32 mmol/L (0.50–1.70); methemoglobin, 38.6%.No significant abnormalities were found in the patient’s brain and chest CT scans on the first day of poisoning. Moreover, symptomatic treatments, including tracheal intubation, ventilator-assisted ventilation, sedation, and intravenous methylene blue injection (20 mg twice daily), along with blood purification, were administered at a local hospital. After treatment, the patient’s consciousness improved. The laboratory test results of Patient 2 are presented in Table 2. On May 11, the toxicology test indicated a DMAC concentration of 0.588 μg/mL in the blood (detection was performed using liquid chromatography-tandem mass spectrometry (LC-MS/MS). The patient also developed hemolytic anemia and liver function impairment. Tracheal intubation was discontinued on the fourth day after poisoning. CT revealed increased fibrous foci in both lungs and new hypostatic inflammation, with no brain abnormalities. He was admitted to our hospital on the seventh day for further treatment.

**TABLE 2:** Laboratory test results of the patient 2 at different time-points following hospital admission.

Biochemical blood indicators	Local hospital				Our department							
	Normal values	Day4	Day5	Day6	Normal values	Day6	Day8	Day12	Day19	Day28	Day60	Day67
WBC (×109/L)	**3.5–9.5**	14.18	14.23	24.30	**3.5–9.5**	13.63	10.62	15.57	8.49	6.74	7.01	6.21
NEU (%)	**40–75**	90.0	88.5		**40–75**	83.50	77.80	85.40	66.10	65.50	52.10	61.00
ALT (IU/L)	**0–41**	117	157	—	**0–35**	147	117	85	32	16	14	12
AST (IU/L)	**0–37**	89	84	—	**14–36**	64	49	29	16	17	19	17
TBIL(μmol/L)	**5.1–19**	21.36	66.64	58.42	**3–22**	40	30.7	12.19	10.8	6.4	7.3	7.0
DBIL (μmol/L)	**1.7–6.8**	9.53	38.18	23.28	**0–5**	0	12.4	5.5	4.2	3.2	2.6	2.3
IBIL (μmol/L)	**2–22**	11.83	28.46	35.14	**0–19**	28	18.3	7.4	6.6	3.2	4.7	4.7
CK (U/L)	**24–190**	122.4	107.2	—	**30–135**	136	129	46	27	48	68	86
CK-MB(ng/mL)	**0–24**	13	16−	—	**0.3–4**	1.90	0.8	0.5	0.6	0.6	0.9	0.9
BUN (mmol/L)	**1.7–8.3**	5.6	11.7	7.0	**2.5–6.1**	9.1	9.56	7.63	5.76	6.11	5.39	4.22
Cr (μmol/L)	**70–115**	51	79	54	**46–106**	68	69	61	72	79	83	75
IL-6(pg/mL)	—	—	—	—	**0–7**	16.9	3.56	3.46	—	2.20	—	
RBC(×10^12^/L)	**4.3–5.8**	3.57	3.53	2.98	**4.3–5.8**	2.99	2.92	2.70	3.25	3.40	4.46	4.17
HGB(g/L)	**130–175**	107	105	82	**130–175**	94.0	91.0	84	105.0	110.0	134.0	126.0
DD-i (μ g/mL)	**0–1**	11.66	—	—	**<0.50**	1.95	0.90	1.12	1.19	0.91	0.38	0.63

WBC, white blood cells; NEU, neutrophils; ALT, alanine transaminase; AST, aspartate aminotransferase; TBIL, total bilirubin; DBIL; direct bilirubin; IBIL; indirect bilirubin; CK, creatine kinase; CK-MB, creatine kinase-MB; BUN, blood urea nitrogen; Cr, creatinine; IL-6, interleukin 6; RBC, red blood cells; HGB, hemoglobin; DD-i, D-dimer.

Values in bold represent the normal values of indicators for different hospitals.

Upon admission, the patient was conscious and cooperative during the physical examination. He exhibited slight yellowing of the skin and mucous membranes, with his pupils measuring approximately 3 mm in diameter with a normal light reflex. His heart rate was 82 beats/min. He was treated with dexamethasone (10 mg), vitamin B_1_, mecobalamine, liver protective agents (polyene phosphatidylcholine), and blood purification. On the 12th day after poisoning, chest CT revealed inflammation in the lower lobes of both lungs. The cerebral MRI revealed slight ischemic degeneration of white matter in both cerebral hemispheres. On the 60th day, follow-up MRI and CT scans showed no significant abnormalities. The electroencephalogram was normal, but electromyography indicated peripheral neuropathy in the upper and lower extremities, primarily affecting motor and sensory functions. Notable findings included decreased conduction velocity in the median motor nerve at the cubital tunnel, reduced amplitude in the left superficial peroneal nerve conduction, absent conduction in the right superficial peroneal nerve, decreased conduction velocity in the left sural nerve, and absent conduction in the left tibial nerve. The patient continued to receive vitamin B_1_, mecobalamine, and other nutritional nerve treatments, with regular follow-up reviews.

## 3 Discussion

The poisoning substances primarily involved DMAC and 4,4′-ODA, which have not been previously reported. When the patients opened the blue sheet of the pipeline, a waste liquid stored in the pipeline at normal temperature was ejected. Following transient exposure to high concentrations of the toxins, the patients exhibited dizziness, unconsciousness, cyanosis of the lips, nail beds, and skin, as well as progressing to a coma. Laboratory examinations showed methemoglobinemia and liver function damage. We speculate that 4,4′-ODA, an aromatic amine compound, was responsible for the methemoglobinemia (National Toxicology Program, 1980). It can cause poisoning through inhalation and skin contact and is known to have irritating, mutagenic, and carcinogenic effects (Hayden et al., 1978). In the body, it oxidizes to produce toxic metabolites. 4,4′-ODA binds to hemoglobin as a diamine or monoacetyl-diamine (Sabbioni and Schütze, 1998). It can lead to acquired methemoglobinemia. The direct oxidation of hemoglobin in the blood to methemoglobin may also interfere with the methemoglobin reduction system in red blood cells (Coleman et al., 1991), resulting in tissue hypoxia, diffuse cyanosis, and hemolytic anemia.

Acquired methemoglobinemia is most commonly caused by various chemical substances or drug poisoning (Dhungel et al., 2021). In this condition, the bivalent iron in the hemoglobin molecule is replaced by trivalent iron, resulting in a loss of the ability to bind oxygen. Common toxic agents include sodium nitrite, aniline, and nitrobenzene. Typically, when methemoglobin concentrations exceed 50%, severe symptoms, such as shock, convulsions, and coma, may manifest. Methylene blue is the primary treatment for reducing methemoglobin levels (Iolascon et al., 2021). Hemolysis associated with methemoglobinemia can be attributed to the toxicity of substances, G6PD deficiency (Schuurman et al., 2009), and methylene blue overdose. In this event, the poison or its metabolites can cause hemolysis by damaging cell membranes or disrupting intracellular enzyme systems (Yu et al., 2022; Shi et al., 2022), resulting in a rapid decline in red blood cell count and hemoglobin levels, leading to hemolytic anemia, alongside significant increases in bilirubin levels and liver function impairment. In Patient 1, hemolytic anemia was particularly severe, and muscle tissue injury was exacerbated by convulsions, resulting in a significant increase in creatine kinase levels. The patient’s condition improved after transfusion, blood purification, and symptomatic treatment. Studies have demonstrated that blood purification can significantly decrease creatine kinase levels in the bloodstream, consequently safeguarding renal function.

In addition, subsequent to discharge, all patients exhibited neurological deficits. Patient 1 exhibited fingertip numbness 16 days after poisoning. The MRI findings revealed a symmetrical distribution of speckle-like and punctate-long T_1_ and T_2_ signals within the internal capsule beneath the cortex of both cerebral hemispheres. T_2_ FLAIR revealed hyperintensity, while DWI indicated elevated signals consistent with toxic encephalopathy. Abnormal signals in the internal capsule have been more frequently observed in patients suffering from carbon monoxide poisoning, heroin encephalopathy, and toluene toxicity. Patient 2 developed peripheral neuropathy 60 days after exposure, predominantly manifesting as motor and sensory deficits. Nevertheless, the exact pathophysiological mechanisms remain elusive. Notably, there is no prior documentation of toxic encephalopathy or peripheral nervous system damage resulting from DMAC and 4,4′-ODA poisoning.

Notably,the *in vitro* cytotoxicity experiments conducted on Hep 3B and IMR 32 human cell lines demonstrated that 4,4′-ODA is capable of inducing apoptosis (Singh et al., 2014). Cases of DMAC poisoning are predominantly reported following prolonged chronic exposure or high-dose incidents, often presenting with significant hepatitis (Wang and Chen, 2020), gastrointestinal symptoms, and central nervous system damage within a 2-month incubation period. Patients with acute severe toxic hepatitis induced by DMAC may experience relapses over short intervals, with liver enzyme levels typically rising. Furthermore, abnormal liver function, accompanied by pathological alterations, often leads to disrupted bilirubin metabolism and jaundice. Dimethylacetamide is mainly metabolized by cytochrome P450 in the liver, and its hepatotoxicity is associated with ROS-induced DNA damage (Perbellini et al., 2003). Neurotoxicity primarily affects the central nervous system. Workers subjected to long-term DMAC exposure frequently report symptoms such as headaches, vertigo, lethargy, hallucinations, depression, and impairments in speech and cognition, which can even progress to coma (Song et al., 2018; Tutkun et al., 2019). These effects may be linked to oxidative stress responses, lipid peroxidation, calcium homeostasis disruption, and apoptosis of target cells, among other factors (Liu et al., 2016).

## 4 Conclusion

In conclusion, DMAC and 4,4′-ODA can be absorbed via the respiratory tract and skin, leading to potential damage to the liver and kidneys, as well as the nervous system. Early and appropriate interventions, such as hemoperfusion and blood purification, should be implemented following diagnosis (Su et al., 2000). Moreover, monitoring the progression of hemolytic anemia and hepatic injury is essential. Measures should be taken to mitigate intracranial edema, alleviate clinical symptoms (Dang et al., 2019), prevent toxic encephalopathy and peripheral nerve damage.

## 5 Recommendations

Our case underscores the necessity for enterprises that employ workers in contact with 4,4′-ODA and DMAC to provide comprehensive occupational health training, enhance awareness of the toxicity of these substances, reinforce safety protocols, and conduct regular health assessments (Li et al., 2022), Encourage enterprises to strengthen preventive measures to reduce the risk of occupational environmental exposure to poisons. For example, in 2024 the JSOH(Japanese Society of Occupational Health) recommended 5 ppm as the occupational exposure limit (OEL) for DMAC. These measures can help prevent the recurrence of similar poisoning incidents.

## 6 Limitations

Limitations of the data presented here, such as the lack of controlled variables or well-defined baselines for assessing the severity of outcomes, will be refined in future work.

## Data Availability

The original contributions presented in the study are included in the article/supplementary material, further inquiries can be directed to the corresponding author.
